# Implications of the 21-gene recurrence score assay (Oncotype DX) on adjuvant treatment decisions in ER-positive early-stage breast cancer patients: experience of Kuwait Cancer Control Center

**DOI:** 10.1186/s43046-020-00025-5

**Published:** 2020-03-04

**Authors:** Salah Fayaz, Heba El-Sayed Eissa, Gerges Attia Demian

**Affiliations:** 1 Department of Radiation Oncology, Kuwait Cancer Control Center, Kuwait; 2 National Cancer Institute, Cairo University, Egypt

**Keywords:** Early breast cancer, Adjuvant systemic treatment, Oncotype DX recurrence score

## Abstract

**Background:**

The Oncotype DX is a quantitative assay of the expression of 16 tumor-related genes and 5 reference genes that predicts the potential of adjuvant chemotherapy benefit in estrogen receptor (ER)-positive early breast cancer patients. The study aims to evaluate the impact of Oncotype DX as a tool for adjuvant treatment decision of ER-positive, HER2-negative, N0/N1 early-stage breast cancer patients and to determine which clinicopathological criteria derived the greatest advantage.

**Results:**

A hundred patients at a median age of 50 years were included. TNM stage distribution was 34, 63, and 3 patients for stages I, II, and IIIA respectively. Fifty-four patients had luminal A and 46 had luminal B tumors. The recurrence score (RS) results were low, intermediate, and high risk in 54, 34, and 12 patients respectively. Before the test results, adjuvant chemoendocrine therapy (CET) was recommended for 46 patients while 54 were advised for endocrine therapy (ET). After getting the test results, 25 patients received CET (1, 12, and12 patients in the low-, intermediate-, and high-risk groups respectively) and 75 received ET. Treatment change was documented in 37 patients (8 patients from ET to CET and 29 from CET to ET; *p* = 0.001, McNemar test). Treatment change was significant among patients ≤ 50 years, luminal B tumors, stage II and IIIA disease, and node-positive disease.

**Conclusion:**

Oncotype DX testing resulted in significant changes in the adjuvant treatment decisions in ER-positive, HER2-negative early breast cancer particularly in the case of young, luminal B, N1, and stage II–IIIA disease.

## Background

The clinicopathologic features have traditionally guided the decision-making of chemotherapy use in the adjuvant sitting of early breast cancer [1]. The most effective chemotherapy regimens offer an average of one third reduction in 10-year breast cancer mortality and 30% relative reduction in the risk of recurrence [2]. Among estrogen receptor-positive (ER-positive), axillary node-negative (N0) patients, this would result in an absolute gain of 5%. Many of these patients would be overtreated if chemotherapy is given on the basis of clinicopathologic features alone and would have been adequately managed with endocrine therapy alone. The recent advances in gene expression profiling of breast tumors have improved the ability to predict a patient’s risk of distant recurrences and likelihood of response to endocrine therapy and/or chemotherapy. The 21-gene recurrence score (RS) assay stratifies ER-positive, HER2-negative patients according to the risk for distant recurrence into low-, intermediate-, and high-risk categories, independent of their clinicopathologic features [3], and predicts the benefit of adjuvant chemotherapy [4, 5].

## Aim of the work

The study was conducted to evaluate the impact of Oncotype DX recurrence score on adjuvant treatment decision of ER-positive, early-stage breast cancer patients to gain insight into the real-world utility of the assay in Kuwait and to determine which clinicopathological criteria derived the greatest advantage.

## Methods

A total of 100 Oncotype DX recurrence score (RS) results were available to our center for ER-positive, HER2-negative, N0/N1 excised invasive breast tumors. The RS were requested on the excised tumor tissues during the period between January 2011 and October 2017. Recruitment of patients for the test was slow in the first 4 years (14 patients) as patients were required to pay out-of-pocket the test cost. However, between 2015 and 2017, more patients (86 patients) had the test as it came to be sponsored by the Ministry of Health.

The decisions to go for Oncotype DX test is made by the multidisciplinary team (MDT) upon having the surgical pathology final report including tumor type, size, grade, estrogen and progesterone receptors (ER and PR), HER2, and nodal status. According to our National Guidelines (https://kuwaitcancercenter.net/Physicians/Guidelines.html), the option of either endocrine therapy (ET) or chemoendocrine (CET) adjuvant treatment is discussed with the patient before the RS test result and the decision is documented taking into account both the patient and oncologist point of view. Once RS is made available, a second meeting is held and final decision is made and recorded taking into consideration the information added by test score.

### Statistical analysis

The primary objective of the study is to assess the proportion of change in the treatment recommendations before and after RS results. The McNemar test is used to assess the association of recurrence score results with the changes in the treatment decisions.

## Results

The clinicopathological characteristics of the studied women are summarized in Table 1. The median age is 50 years (range 38–74). The majority (94%) had ductal histology. The tumor was resected in 78% by wide excision and in 22% by mastectomy. Axillary sentinel lymph node (SLN) biopsy was the form of axillary staging in 84% and axillary clearance in 16%. Median tumor size was 2.2 cm (range 0.7–7 cm). Seventy-six percent were node negative (N0), 10% showed microscopic metastasis (N1mic), and 14% had positive lymph nodes (N1). Median number of positive nodes was 1 (range 1–3). TNM stage distribution was 34%, 63%, and 3% for stages I, II, and III respectively.

**Table 1:** Patients, tumor, and treatment characteristics of 100 patients who had Oncotype DX recurrence score assessment

			Recurrence score risk category						*p* value
	Whole group		Low Risk		Intermediate risk		High risk		
	*n*	%	*n*	%	*n*	%	*n*	%	
All patients	100	100	54	54	34	34	12	12	
Age									
Age ≤ 50 years	51	51	30	55.6	16	47.1	5	41.7	0.583
Age > 50 years	49	49	24	44.4	18	52.9	7	58.3	
Menopausal status									
Premenopausal	59	59	35	64.8	19	55.9	5	41.7	0.304
Postmenopausal	41	41	19	35.2	15	44.1	7	58.3	
Tumor histological grade									
Grade 1	15	15	11	20.4	4	11.8	0	0	0.003
Grade 2	64	64	32	59.3	27	79.4	5	41.7	
Grade 3	17	17	9	16.7	2	5.9	6	50	
Grade (unknown)	4	4	2	3.7	1	2.9	1	8.3	
Ki-67 index									
Ki-67 ≤ 15	51	51	37	68.5	13	38.2	1	8.3	< 0.001
Ki-67 > 15	38	38	11	20.4	17	50	10	83.3	
Ki-67 (unknown)	11	11	6	11.1	4	11.8	1	8.3	
Tumor type									
IDC	94	94	52	96.3	30	88.2	12	100	0.195
ILC	6	6	2	3.7	4	11.8	0	0	
Primary tumor surgical approach									
WLE	78	78	39	72.2	29	85.3	10	83.3	.316
Mastectomy	22	22	15	27.8	5	14.7	2	16.7	
Axillary surgical management									
SLN	84	84	46	85.2	28	82.4	10	83.3	0.938
Axillary clearance	16	16	8	14.8	6	17.6	2	16.7	
pT stage									
pT1	39	39	24	44.4	13	38.2	2	16.7	0.381
pT2	55	55	26	48.1	20	58.8	9	75	
pT3	6	6	4	7.4	1	2.9	1	8.3	
pN stage									
pN0	76	76	42	77.8	25	73.5	9	75	0.987
pN1mic	10	10	5	9.3	4	11.8	1	8.3	
pN1	14	14	7	13	5	14.7	2	16.7	
UICC-AJCC TNM stage									
Ia	31	31	18	33.3	11	32.4	2	16.7	0.719
Ib	3	3	3	5.6	0	0	0	0	
IIa	47	47	24	44.4	16	47.1	7	58.3	
IIb	16	16	8	14.8	6	17.6	2	16.7	
IIIa	3	3	1	1.9	1	2.9	1	8.3	
Lymphovascular invasion									
LVI+	17	17	5	9.3	10	29.4	2	16.7	0.046
LVI−	78	78	46	85.2	22	64.7	10	83.3	
LVI-unknown	5	5	3	5.5	2	5.9	0	0	
Luminal tumor type									
Luminal A	54	54	36	66.7	16	47.1	2	16.7	0.004
Luminal B	46	46	18	33.3	18	52.9	10	83.3	
Estrogen receptor positivity percentage									
ER ≥ 90%	88	88	49	90.7	29	85.3	10	83.3	0.648
ER 30–80	12	12	5	9.3	5	14.7	2	16.7	
Progesterone receptor positivity percentage									
PR ≥ 90%	48	48	32	59.3	14	41.2	2	16.7	0.017
PR 0–85%	52	52	22	40.7	20	58.8	10	83.3	
Treatment recommendation before RS assessment									
Chemoendocrine recommendation	46	46	18	33.3	17	50	11	91.7	< 0.001
Endocrine only recommendation	54	54	36	66.7	17	50	1	8.3	
Treatment recommendation after RS assessment									
Chemoendocrine recommendation	25	25	1	1.9	12	35.3	12	100	< 0.001
Endocrine only recommendation	75	75	53	98.1	22	64.7	0	0	
Type of hormonal treatment given									
Tamoxifen	58	58	36	66.7	17	50	5	41.7	
Aromatase inhibitors	42	42	18	33.3	17	50	7	58.3	

*IDC* invasive duct carcinoma, *ILC* invasive lobular carcinoma, *WLE* wide local excision, *SLN* sentinel lymph node

RS results were low, intermediate, and high risk in 54, 34, and 12 patients respectively. Before the test results, the multidisciplinary team recommended adjuvant CET in 46 patients and ET alone in 54 patients based on the clinicopathological criteria (Table 2). After getting the test results, 25 patients received CET (1, 12, and12 patients in the low-, intermediate-, and high-risk groups respectively) and 75 received ET. Treatment was changed in 37 patients (37%) after RS was made available (*p* = 0.001, McNemar test). In 29 of the 46 patients (63%) who were recommended CET, treatment was revised to ET alone, and in 8 of the 54 (14.8%), adjuvant therapy was changed from ET to CET. The overall reduction in chemotherapy recommendation was met in 21 women (21%). Among the 54 patients proved to be low risk (RS < 18), 18 were initially recommended chemotherapy (of whom only one received CET) and 36 were recommended endocrine treatment (none received chemotherapy). For the 34 intermediate-risk (RS ≥ 18 and < 30) women, 17 were recommended CET (of them 5 received) and 17 ET alone (7 received CET). Among 12 high risk (≥ 31) patients, 11 were initially advised for CET and only 1 was advised for ET alone (all received chemotherapy).

**Table 2:** Clinicopathological characteristics of patients according to their initially suggested treatment

			Initially proposed treatment groups				*p* value
	Whole group		ET		CET		
	*n*	%	*n*	%	*n*	%	
All patients	100	100	54	54	46	46	
Age							
Age ≤ 50 years	51	51	27	50	24	52.2	0.829
Age > 50 years	49	49	27	50	22	47.8	
Menstrual status							
Premenopausal	59	59	32	59.3	27	58.7	0.955
Postmenopausal	41	41	22	40.7	19	41.3	
Tumor histological grade							
Grade 1	15	15	10	18.5	5	10.9	0.004
Grade 2	64	64	39	72.2	25	54.3	
Grade 3	17	17	3	5.6	14	30.4	
Grade (unknown)	4	4	2	3.7	2	4.3	
Ki-67 index							
Ki-67 ≤ 15	51	51	33	61.1	18	39.1	< 0.001
Ki-67 > 15	38	38	10	18.5	28	60.9	
Ki-67 (unknown)	11	11	11	20.4	0	0	
Tumor type							
IDC	94	94	53	98.1	41	89.1	0.060
ILC	6	6	1	1.9	5	10.9	
Primary tumor surgical approach							
WLE	78	78	43	79.6	35	76.1	.424
Mastectomy	22	22	11	20.4	11	23.9	
Axillary surgical management							
SLN	84	84	46	85.2	38	82.6	0.349
Axillary clearance	16	16	8	14.8	8	17.4	
pT stage							
pT1	39	39	29	53.7	10	21.7	< 0.001
pT2	55	55	25	46.3	30	65.2	
pT3	6	6	0	0	6	13	
pN stage							
pN0	76	76	49	90.7	27	58.7	0.001
pN1mic	10	10	5	9.3	5	10.9	
pN1	14	14	0	0	14	30.4	
Lymphovascular invasion							
LVI+	17	17	3	5.6	14	30.4	0.001
LVI−	78	78	50	92.6	28	60.9	
LVI-unknown	5	5	1	1.9	4	8.7	
Luminal tumor type							
Luminal A	54	54	40	74.1	14	30.4	< 0.001
Luminal B	46	46	14	25.9	32	69.6	
Estrogen receptor positivity percentage							
ER ≥ 90%	88	88	48	88.9	40	87.0	0.295
ER 30–80	12	12	6	11.1	6	13.0	
Progesterone receptor positivity percentage							
PR ≥ 90%	48	48	28	51.9	20	43.5	0.406
PR 0–85%	52	52	26	48.1	26	56.5	

*IDC* invasive duct carcinoma, *ILC* invasive lobular carcinoma, *WLE* wide local excision, *SLN* sentinel lymph node

Treatment change was significant among patients ≤ 50 years, luminal B tumors, stage II and IIIA disease, and node-positive disease (Table 3).

**Table 3:** Summary of the treatment change among different subgroups of the 100 patients who had Oncotype DX recurrence score assessment

		Treatment given				Exact sig.
		Endocrine		Chemoendocrine		
		*n*	%	*N*	%	
Treatment Recommendation before RS	Whole group					
	Endocrine	46	46	8	8	0.001
	Chemoendocrine	29	29	17	17	
	Luminal A					
	Endocrine	32	59.3	8	14.7	0.648
	Chemoendocrine	11	29.4	3	5.6	
	Luminal B					
	Endocrine	14	30.4	0	0	< 0.001
	Chemoendocrine	18	39.2	14	30.4	
	≤ 50 years					
	Endocrine	25	49.1	2	3.9	0.002
	Chemoendocrine	15	29.4	9	17.6	
	> 50 years					
	Endocrine	21	41.9	6	12.2	0.115
	Chemoendocrine	14	28.6	8	16.3	
	N0					
	Endocrine	42	55.2	7	9.2	0.093
	Chemoendocrine	16	21.1	11	14.5	
	N1					
	Endocrine	4	16.7	1	4.2	0.002
	Chemoendocrine	13	54.1	6	25	
	Stage I					
	Endocrine	24		5		1.0
	Chemoendocrine	4		1		
	Stages II and III					
	Endocrine	22		3		< 0.001
	Chemoendocrine	25		16		

The definition of the RS risks group was re-defined in concordance with the recently published TailorX study [6], into two groups (low or high) with a cutoff modulated by clinicopathological risk for the patients ≤ 50 years. Patients aged > 50 years with RS ≤ 25 and ≤ 50years with RS < 16 are considered for ET while those patients < 50 years with RS ≥ 16 and > 50 years with RS > 25 are considered for CET. As the TailorX study included only axillary node-negative patients, the re-analysis included the 76 node-negative patients. Should the results be available, the treatment would have been changed in 18 patients (24%): from CET to ET in 9 and from ET to CET in 9.

The median follow-up was 12 months (3–75 months). One patient among the low risk group had a systemic relapse in the bone after 30 months of adjuvant tamoxifen. Another developed contralateral breast cancer after 2 years of adjuvant letrozole, likely a second primary cancer.

## Discussion

In the era of personalized medicine, the “one size fits all” model is no longer attractive. The Oncotype DX assay which is a quantitative analysis of gene expression assessing the expression of 16 tumor-related genes and 5 reference genes is an excellent example of the personalized medicine. It is not merely a prognostic tool but more importantly predicts the potential of chemotherapy responsiveness. In the prospective confirmatory trial involving 10,253 women with HR-positive, HER2-negative, axillary node-negative breast cancer, 1626 women (15.9%) who had a recurrence score of 0 to 10 were assigned for endocrine therapy alone. The 5-year distant recurrence-free survival was 99.3%, and the overall survival was 98.0% [7]. Also, among 6711 women with a recurrence score of 11 to 25 who were randomized to receive either ET or CET, ET proved to be noninferior regarding invasive disease-free survival at 9 years (83.3% for ET vs. 84.3% for CET), distant recurrence-free survival (94.5% vs. 95.0%), and overall survival (93.9% vs. 93.8%). Some benefit of chemotherapy was seen in women ≤ 50 years of age having a recurrence score of 16 to 25 (TailorX study) [6].

In the present study, Oncotype DX assay has significantly impacted the prescription of chemotherapy. Of 46 patients recommended for CET therapy, 29 (63%) changed to ET alone sparing a group of patients the toxicity as well as the economic impact of chemotherapy. Even more importantly, 8 out of 54 patients (14.8%) of patients who were advised for ET were prescribed CET therapy following Oncotype DX testing. These patients are the most likely to get benefit from the test as they offered a treatment which might significantly reduce their risk of relapse.

The overall change of treatment recommendations was documented in 37% of our N0/N1 patients. The UK figure was 27% [8]; Germany, 33% [9]; Spain, 32% [10]; and France, 34% [11]. The treatment change was 32% in a pooled meta-analysis of the previous European studies. In Ontario, Canada, the percentage was 38% [12]; Mexico, 32% [13]; Japan, 38% [14]; Hong Kong, 23.3% [15]; and United Arab Emirates, 27.7% [16].

The variation of change in treatment recommendation is likely related to the proportion of patients who had a pre-test CET recommendation which is a sequence of the clinicopathological risk factors like age, menopausal status, tumor grade, lymph node status, and Ki-67 proliferative index. In the Japanese study as well as this study, N1 patients were included thus boosting the initial CET advise. In node-positive patients, a rate of treatment change of 51% was reported in 138 retrospectively studied patients [17].

In this study, a strong association was shown between RS and clinicopathological factors like tumor grade, Ki-67 index, and luminal tumor type. No grade I tumors had a high RS. Similarly, in a study from Ontario, no high RS tumors were grade I in 1000 analyzed patients. These data suggest that such factors might serve as a tool to select patients for whom the expensive RS test can be skipped. For instance, if the clinicopathological factors predicted a low probability for high RS (which is the sole factor to consider chemotherapy), Oncotype DX might be withdrawn. Furthermore, treatment changes were more significant among younger, N1, luminal B, and more than stage 1 disease in whom the change is more likely from CET to ET; thus, patients can be prioritized in case of financial restrictions.

## Conclusion

In conclusion, the use of the Oncotype DX assay led to significant changes in the adjuvant treatment decisions in ER-positive, HER2-negative, early breast cancer. Ultimately, the test resulted in a net reduction in treatment recommendations for adjuvant chemotherapy particularly in young patients, luminal B tumors, N1 disease, and stage II to IIIA disease.

## Data Availability

The datasets used and analyzed during the current study are available from the corresponding author on reasonable request.

## Abbreviations

CET: Chemoendocrine therapy
ER: Estrogen receptor
ET: Endocrine therapy
Her 2: Human epidermal growth factor receptor 2
N0: Lymph node negative
N1mic: Microscopic metastasis in lymph nodes
RS: Recurrence score
